# Sweat Producing Agents

**Published:** 1879-07

**Authors:** 


Sweat Producing Agents.—The following is an extract taken from a selection of the *Cincinnati Lancet and Clinic*:—According to Luchsinger (*Pflüger's Arch. XIV. S.* 375) nicotine can be looked upon as an agent through which the sweat centers may be excited. He found by further trial that nicotine, in rare cases, causes the secretion of sweat to a slight extent, even after the division of the sciatic nerve. Calabar bean exhibited more activity in this particular. He observed a slight though constant secretion on the affected side when the solution of physostigmin was injected directly under the skin of the foot. The very important difference in secretion between the two sides show, however, that a great central excitement follows slight peripheral excitations, or frequently supervenes when the latter are wanting.

Marme (Nachrichten der Gottinger Ges. der Wissensch, 1858, No. 3) made observations on the action of camphor and liquor ammoniæ acetatis on the secretion of sweat. He injected these agents (the camphor being dissolved in oil), and the animals experimented on began to sweat throughout the body. After division of the sciatic nerve on one side, the so-called hydrogogues failed to produce sweat on the foot operated on.

In cats, whose spinal cord was divided at the ninth dorsal vertebra, Marme could not produce any secretion of sweat with subcutaneous injections of camphor.

Luchsinger (Pflüger's Arch. XVI., S. 538) advises the skeptics, who cannot be convinced of the existence of his spinal sweat centers, to perform the following experiments with picrotoxin :

Tracheotomy is to be performed in a cat, artificial respiration established, the spinal cord to be separated from the medulla at the atlas, one of the sciatic nerves to be divided, and a few cubic centimetres of a solution of picrotoxin injected subcutaneously. After a few minutes convulsions supervene, and at the same time a profuse secretion of sweat occurs in all the feet, except the one operated on, which remains entirely dry.

J. Ott and Wood Field (Jour. of Physiol. I. P., 193) tried the action of muscarin on the secretion of sweat. They found that this agent produced sweating in the foot even after division of the sciatic nerve of the corresponding side, and this was arrested by subsequent injections of atropine.

So far as the action of muscarin is concerned, my experiments on cats gave about the same result as those of Ott and Field. Instead of muscarin I used extracts of the poisonous mushroon (*agaricus muscarins*). The dried and powdered fungus was treated with strong alcohol, evaporated to dryness, and the residue desolved in water. This was filtered and evaporated in vacuo and over sulphuric acid to the consistency of the syrup. If the sciatic nerve or the spinal cord in a suitable place is divided, and three centigrammes of the above extract dissolved in one cubic centimetre of water is injected into the jugular vein, profuse sweating sets in either immediately after or in the course of a minute in all four extremities. There occurs simultaneously a profuse secretion of saliva and tears. Hence, muscarin acts in the same manner as pilocarpin, by exciting the peripheral ends of the nerves of sweating.

Much more important to me were such agents as cause sweating by exciting the sweat centre. In this class I reckon ammonium aceticum, physostigmium sulphuricum, nicotin and picrotoxin. All my experiments were made on sweating and slightly curarized animals, and the drug was thrown into the jugular vein. One experiment with division of the spinal cord at the first cervical vertebra was followed by a second in which the cord was left intact.

I think my observations will be better appreciated by giving two such parallel experiments out of every series.

I. *Ammonium Aceticum*, 10 *p c solution.*

January 18th, 1870. A profusely sweating cat was curarized, and the spinal cord divided at the first cervical vertebra. The paws were dry. At 1.25 P.M., three centigrammes of ammonium aceticum were injected. At 1.30 P.M., and longer, all four paws were dry.

January 18th, 1879. A sweating cat was curarized, and at 1.50 P.M., the paws were perfectly dried. They remained dry until 2 P.M., at which moment an injection of three centigrammes of solution of ammonium aceticum was made. At 2—3 P.M., all four paws were richly covered with sweat.

II. *Physostigminum Sulphuricum.*

August 24th, 1878. A profusely sweating cat was curarized, and the spinal cord at the first cervical vertebra and the left sciatic nerve divided. The paws were dry. At 12.35 P.M., an injection of 4 milligrammes of physostigmin was made. At 12 45 all the paws were dry. The left sciatic was then excited with induced electricity, and a minute afterwards the left hind paw was richly covered with sweat.

August 24th, 1878. A small cat sweating on its fore paws was curarized and the right sciatic divided. At 10.29 the paws were carefully dried. Till 10.37 no secretion of sweat. At 10.38 an injection of 4 milligrammes of physostigmin was made; and at 10.39 both four paws and the left hind one were richly covered with drops of sweat. The paws being dried off, were immediately covered again with sweat. At 10.52 the same was repeated, but during the whole time the right hind foot remained dry.

III. *Nicotin.*

August 21st, 1878. A profusely sweating cat was slightly curarized, the spinal cord at the first cervical vertebra and the right sciatic nerve divided. The sweating stopped. At 11.29 a drop of nicotin in two centimeters of water was injected. Along with general spasm there appeared a small quantity of sweat on all except the hind paw. After dying no new sweat appeared, the paws remaining dry till 11.40. At 11.43 the trachea was held for a space of two minutes, but in spite of general convulsions there was no secretion of sweat. At 11.50 a drop of nicotin was injected. Clonic spasm of the left hind leg followed ; but up to 11.57 there was no secretion of sweat.

August 21st, 1878. A profusely sweating cat was curarized, and the left sciatic divided. The paws remained dry. At 1.7 a drop of nicotin was injected. At 1.8 the two fore paws and right hind paw were covered with sweat. As often as the three named paws were dried they were covered again with drops of sweat. The left hind paw remained dry all this time.

